# From Stress to Sick(le) and Back Again–Oxidative/Antioxidant Mechanisms, Genetic Modulation, and Cerebrovascular Disease in Children with Sickle Cell Anemia

**DOI:** 10.3390/antiox12111977

**Published:** 2023-11-07

**Authors:** Marisa Silva, Paula Faustino

**Affiliations:** 1 Departamento de Genética Humana, Instituto Nacional de Saúde Doutor Ricardo Jorge (INSA), Av. Padre Cruz, 1649-016 Lisboa, Portugal; marisa.silva@insa.min-saude.pt; 2 Grupo Ecogenética e Saúde Humana, Instituto de Saúde Ambiental (ISAMB), Faculdade de Medicina, Universidade de Lisboa, Av. Prof. Egas Moniz, 1649-028 Lisboa, Portugal; 3 Laboratório Associado TERRA, Faculdade de Medicina, Universidade de Lisboa, Av. Prof. Egas Moniz, 1649-028 Lisboa, Portugal

**Keywords:** oxidative stress, sickle cell anemia, vasculopathy, cerebrovascular disease, antioxidant mechanisms, genetic modulators

## Abstract

Sickle cell anemia (SCA) is a genetic disease caused by the homozygosity of the *HBB*:c.20A>T mutation, which results in the production of hemoglobin S (HbS). In hypoxic conditions, HbS suffers autoxidation and polymerizes inside red blood cells, altering their morphology into a sickle shape, with increased rigidity and fragility. This triggers complex pathophysiological mechanisms, including inflammation, cell adhesion, oxidative stress, and vaso-occlusion, along with metabolic alterations and endocrine complications. SCA is phenotypically heterogeneous due to the modulation of both environmental and genetic factors. Pediatric cerebrovascular disease (CVD), namely ischemic stroke and silent cerebral infarctions, is one of the most impactful manifestations. In this review, we highlight the role of oxidative stress in the pathophysiology of pediatric CVD. Since oxidative stress is an interdependent mechanism in vasculopathy, occurring alongside (or as result of) endothelial dysfunction, cell adhesion, inflammation, chronic hemolysis, ischemia-reperfusion injury, and vaso-occlusion, a brief overview of the main mechanisms involved is included. Moreover, the genetic modulation of CVD in SCA is discussed. The knowledge of the intricate network of altered mechanisms in SCA, and how it is affected by different genetic factors, is fundamental for the identification of potential therapeutic targets, drug development, and patient-specific treatment alternatives.

## 1. Introduction

The hemoglobin (Hb) disorders (or hemoglobinopathies) are the most common genetic diseases worldwide. Sickle cell disease (SCD), in particular, affects about 70% of the 300,000–500,000 children born yearly with a hemoglobinopathy [1,2]. The underlying genetic defects affect Hb function, stability, or Hb levels. Furthermore, Hb abnormalities also lead to changes in the biomechanical properties of red blood cells (RBCs) [3]. The global public health burden of the disease is significant, and even though an increasing proportion of affected children now survive past five years of age, the risk of premature death remains especially high in low-income countries [1,4]. More than just a Hb disorder, its marked clinical heterogeneity renders it a disease spectrum. Different levels of anemia, intra- and extravascular hemolysis, vascular obstruction, inflammation, and metabolic and endocrine alterations, such as, growth failure, osteopenia, hypogonadism, hypothyroidism, and insulin resistance, may be observed in these patients [5,6]. The clinical manifestations of the disease range from very mild to extremely severe and life threatening (e.g., stroke, pulmonary hypertension, acute chest syndrome).

**Figure 1:**
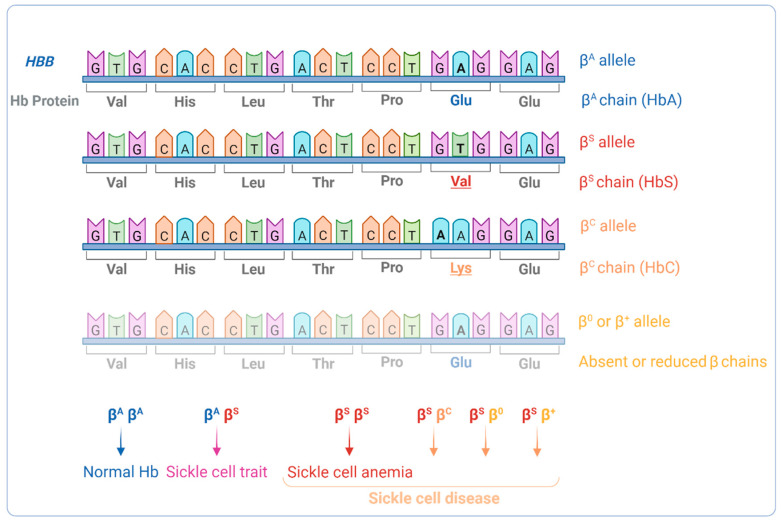
β-globin genotypes and the respective β-globin alterations in sickle cell disease. Partial *HBB* gene and protein sequences are shown. The β^S^ allele results from an A to T mutation in the 6th triplet of the *HBB* gene. This causes substitution of a glutamic acid residue to a valine residue in the 6th position of the mature β-globin chain and gives rise to the production of hemoglobin S (HbS). Mutation in both *HBB* alleles, whether in homozygosity or in compound heterozygosity, results in sickle cell disease (SCD). When the β^S^ allele is present in homozygosity sickle cell anemia (SCA), the most severe form of SCD, arises.

**Figure 2:**
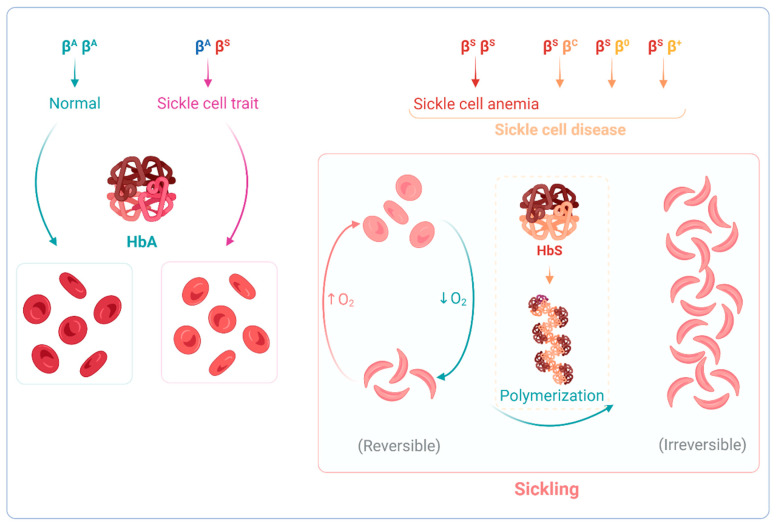
Hemoglobin and red blood cell changes resulting from the different genotypes in sickle cell disease. Heterozigosity for the normal β-globin and the β^S^ alleles underlies a condition called sickle cell trait, which is mostly an asymptomatic carrier state. Compound heterozygosity of β^S^ and other β allele mutation leads to HbS production. HbS has a lower O_2_ affinity and tends to polymerize into rigid fibers inside red blood cells (RBC), under hypoxic conditions. This HbS polymerization leads to RBC sickling due to distortion, increased rigidity, and fragility. Initially, sickling is a reversible process occurring in cycles of oxygenation and deoxygenation. Increased and continuous oxy–deoxy cycles lead to irreversibly sickled RBCs, the hallmark of sickle cell disease.

As with the phenotypes, the genotypes are also varied. First described by James Herrick, in 1910, as the presence of abnormally crescent and sickle-shaped red cells in a blood smear [7], the term SCD currently applies to a group of hemoglobinopathies caused by the presence of the β^S^ allele–an A to T missense mutation in the β-globin gene (*HBB:*c.20A>T). This can occur in homozygosity β^S^β^S^—as in sickle cell anemia (SCA)—or compound heterozygosity of β^S^ with a another mutant *HBB* allele, which either changes the β-globin chain of Hb or reduces *HBB* expression (Figure 1). Whichever the disease-causing genetic change, all patients share the same biochemical phenotype—the presence of the abnormal Hb variant S (HbS). HbS has a single amino acid difference from the normal Hb in the sixth position of the β-globin chain, a substitution of a glutamic acid by a valine (Glu6Val), that changes the protein’s properties and impacts RBC conformation [8] (Figure 1 and Figure 2). Substitution of the same glutamic acid by lysine (Glu6Lys) gives rise to another Hb variant, HbC, which, when inherited concomitantly with HbS, results in HbSC disease, a less severe form than SCA. HbS/β-thal disease is yet another form of SCD, arising from co-inheritance of β-thalassemia (β^0^-thal or β^+^-thal) and HbS.

Although SCD is a single gene disease, its clinical heterogeneity, with the involvement of multiple organs, different degrees of severity, and of environmental factors, mimics a multifactorial mode of genetic transmission. Since patients may have a combination of complications and differ from each other in disease severity, age of onset, and in rate of progression, this translates into a high variability in health-related quality of life and life expectancy [5,9,10]. The overall heterogeneity seems to result from modifying factors, ranging from environmental and sociodemographic factors (discrepancies between patients in high- and low-income settings are apparent), to genetic modulators.

Several interdependent mechanisms have been shown to contribute to vascular pathology, or vasculopathy, in SCD, occurring alongside, or as a result, of one another. These include endothelial dysfunction, cell adhesion, inflammation, chronic hemolysis, ischemia-reperfusion (I/R) injury, vaso-occlusion, and oxidative stress. Current therapies, like bone marrow transplant (curative) and gene therapy (under development), while promising, are hardly available to a high number of patients, particularly in low-income settings.

Oxidative stress, as defined by an imbalance between levels of reactive oxygen species (ROS), reactive nitrogen species (RNS), and activity/concentration of antioxidants, has been extensively reviewed in SCD [5,11,12,13]. However, it is important to understand its role in vasculopathy, as well as the interplay with other disease mechanisms and antioxidant defense, especially in cerebrovascular disease (CVD), which has a significant impact particularly in pediatric patients. The knowledge of this intricate network of mechanisms, and how it is affected by different genetic factors, is fundamental for the identification of potential therapeutic targets, drug development, and patient-specific treatment alternatives.

This review highlights the role of oxidative stress in the pathophysiology of pediatric CVD, a high impact manifestation in children with SCA. Since oxidative stress is an interdependent mechanism in vasculopathy, occurring alongside (or as result of) endothelial dysfunction, cell adhesion, inflammation, chronic hemolysis, I/R injury, and vaso-occlusion, a brief overview of the main mechanisms involved is included. Furthermore, the role of genetic modulators on CVD severity is also discussed.

## 2. Oxidation in RBCs, Hemoglobin, and the Vascular Milieu in SCA

Even though SCA is the most common and severe form of SCD, the compound heterozygous genotypes also lead to the production of sufficient HbS to promote intracellular RBC sickling [14]. In those individuals, the majority of Hb is HbS, whereas in individuals with sickle cell trait (heterozygotes for the abnormal HbS), HbA is the main form present (Figure 1).

Physiologically, oxygenated Hb (ferrous state) is relatively stable. However, it can autoxidize to methemoglobin (metHb) (ferric state), particularly in low-oxygen-saturation environments, like in the microcirculation. ROS production in RBCs is almost exclusively a result of Hb autoxidation, and it is even more pronounced for unstable hemoglobins like HbS, which, contrary to HbA, has a low oxygen affinity [15]. Consequently, in SCD, Hb autoxidation is more pronounced than in normal physiological conditions, promoting a marked pro-oxidant vascular environment, especially in the microcirculation [16]. Under those hypoxic conditions, HbS polymerizes into long rigid fibers inside RBCs. Each of these fibers consists of seven intertwined double strands (with cross-linking) that distort and damage the membrane and cytoskeleton of RBCs [17]. The distortion and damage culminates in sickling, where RBCs adopt a characteristic crescent, or sickle, shape (Figure 2). The subsequent alterations in microrheological, as well as in biomechanical, properties affect their aggregability, deformability, and cell adhesion [17]. The shape changes are initially reversible, occurring in cycles of oxygenation and deoxygenation (oxy–deoxy). As the number of oxy–deoxy cycles increases, the altered RBCs become irreversibly sickled (SSRBC), a state associated with increased fragility, shorter lifespan, and higher propensity to adhere to the vessel wall [18].

Being more fragile, SSRBCs rupture easily (intravascular hemolysis) with consequent release of Hb and heme. Furthermore, extravascular hemolysis is exacerbated due to phagocytosis of SSRBCs by spleen and liver macrophages. Both types of hemolysis contribute to a reduction in the SSRBCs’ lifespan (one week, contrasting with 120 days for normal RBCs), increasing anemia and stimulating production, and an excess of circulating immature RBCs, or reticulocytes (also called stress reticulocytes). Compromised SSRBCs deformability, which impairs their normal flow through capillaries, contributes to vaso-occlusion. This process is complex and may involve multistep multicellular interactions that include endothelial activation, recruitment of adherent leukocytes, interactions of SSRBCs with adherent neutrophils, and vascular obstruction by heterotypic multicellular aggregates [19]. The overall process leads to vaso-occlusion, which in turn culminates in cumulative organ damage, and results in life-threatening crises [19].

**Figure 3:**
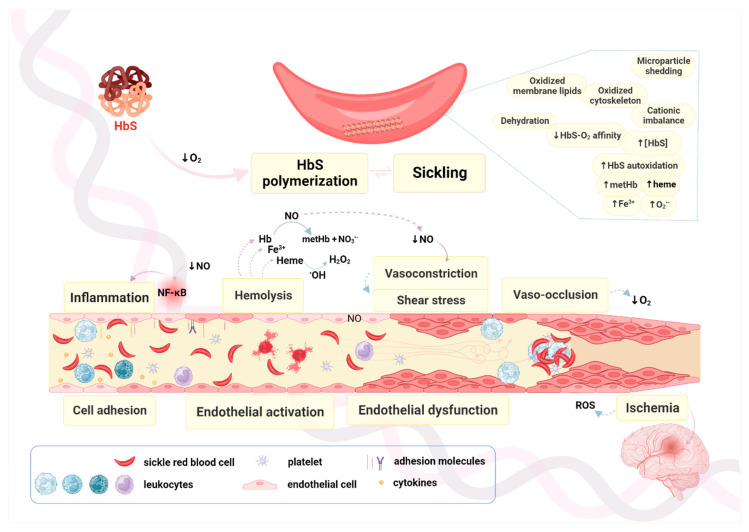
Oxidative mechanisms in the pathobiology of sickle cell disease. Oxidative stress in SCD is not an isolated mechanism. Several pathobiological mechanisms unfold inside blood vessels, especially in those with lower oxygen pressure, like arterioles, capillaries, and post-capillary venules. Those mechanisms range from hemolysis to vaso-occlusion, culminate in ischemia, and ultimately, in tissue damage. Hemoglobin S (HbS) damages and causes membrane dysfunction on the sickle red blood cell (SSRBC) membrane, which leads to hemolysis. Oxidized membrane proteins expose phosphatidylserine. SSRBCs rupture and their content is released into the circulation through the intravascular hemolysis. This results in NO scavenging by cell-free Hb, enhanced by depletion of L-arginine, the nitric oxide synthases’ (NOS) substrate, and asymmetric dimethylarginine (ADMA) NOS inhibition. Reactive oxygen and nitrogen species (ROS and RNS, respectively) also deplete NO even further. The overall decrease in NO content elicits vasoconstriction which, together with endothelial proliferation, leads to vascular remodeling. Decreased NO and adenine dinucleotides levels lead to activation of platelets and blood clotting factors. Hemolysis also elicits activation of the innate immune system through heme release and other damage-associated molecular pattern (DAMP) molecules. Leukocytes are activated to release inflammatory cytokines which results in inflammation and activation of endothelial cells (EC). Enhanced circulating blood cells’ adhesion to each other promotes formation of multicellular aggregates. This blood cell adhesion, together with adhesion to the activated endothelium, strongly contributes to vaso-occlusion. While vasoconstriction increases blood flow velocity downstream from the constriction site, enhances shear stress, and further contributes to endothelial activation and dysfunction, vaso-occlusion causes flow blockage. The blockage ultimately results in (transient or permanent) ischemia and end-organ damage. Ischemic events are one of the main causes of cerebrovascular disease, namely silent cerebral infarction and stroke, in children with SCD. The interplay of all these mechanisms underlies the clinical manifestations of SCD, the severity of which may be modulated by variants in genes other than *HBB*. HbS: hemoglobin S; metHb: methemoglobin; NF-kB: nuclear factor kappa B.

Inflammation, and chronic hemolysis, particularly intravascular hemolysis, with the release of Hb and heme and the subsequent decrease in nitric oxide (NO) bioavailability, as well as oxidative stress, strongly contribute to vasculopathy. NO reduction shifts the balance towards vasoconstriction (Figure 3), while the activated endothelium expresses several cell adhesion molecules (CAM) like vascular cell adhesion molecule 1 (VCAM-1), intercellular cell adhesion molecule (ICAM), and/or selectins, and undergoes cell proliferation, which additionally contributes to the vaso-occlusive process [20].

These processes work in a self-sustained cycle of repeated cell activation, cell adhesion, inflammation, hemolysis, oxidative stress, vaso-occlusion, and I/R injury, ultimately leading to vasculopathy. Therefore, SCD, and particularly SCA, may be considered a vascular disease with complex pathways that can be specific to each affected organ [11].

## 3. The Vascular Endothelium, Endothelial Activation and Endothelial Dysfunction

Endothelial cells (EC) provide the coating (endothelium) of the luminal surface of all the vessels that constitute the elaborate circulatory network of the body. More than a passive barrier, this interface between the blood and underlying tissues plays an essential role in several cardiovascular functions, including regulation of the vascular tone and growth, fluid and solute exchange, inflammatory response, hemostatic balance, platelet leukocyte interaction, cell proliferation, and angiogenesis [21,22,23,24]. The endothelium’s sensory and effector capabilities allow it to respond to humoral, neural, and mechanical stimuli through the synthesis and release of vasoactive substances [23,24,25]. Vascular dilation occurs as a response to endothelium-derived relaxing factors (EDRFs), like NO and endothelial-derived hyperpolarizing factors (EDHFs) [26,27,28,29]. Vascular contraction, on the other hand, may be induced by the release of endothelium-derived contracting factors (EDCFs), such as endothelin, thromboxane A2, angiotensin II, and superoxide anion (O_2_^•−^) [30,31,32,33]. ECs may also produce growth inhibitors or promoters, such as heparin, and heparin sulfates, platelet derived growth factors, and thrombospondin. NO, endothelin, and angiotensin may also affect vascular growth regulation [34,35].

Although the endothelium is a highly heterogeneous tissue that varies in structure and function, space and time, health and disease, the basic functions of ECs are fundamentally the same regardless of vessel size. They provide a non-thrombogenic surface that prevents blood cells from adhering; mediate the passage of nutrients and other solutes from the blood to the tissues; produce vasoactive agents that maintain vessel patency and prevent platelet aggregation; and keep the vessel lumen open by growing as a monolayer firmly adherent to the basal membrane of the vessel wall [36,37]. On the other hand, ECs differ in morphology, mediator release, antigen presentation, or stress response, and individual cells may differ from the immediately adjacent endothelium [21]. ECs from large and small vessels differ in morphology, with large arteries and veins showing a tightly packed layer of polygonal ECs, while small capillaries and venules consist mainly of individual cylindrical ECs through which the blood cells pass in single file. Moreover, large and small vessels differ in specific markers [36,38].

Both endothelial layers—the glycocalyx and the endothelial cell layer (ESL)—are involved in blood–tissue interactions and consequently in several pathophysiological mechanisms. These include, among others, mechanical stress on blood cells, blood cell/endothelium interaction, and inflammation [21]. Conversely, several physiological, pathological, and therapeutic processes, such as oxidized low-density lipoproteins, growth factors, hypoxia, I/R, changes in plasma composition, or enzyme degrading glycocalyx or ESL components may alter the thickness, composition, and integrity of the ESL [21].

Hence, the balance between endothelial injury and recovery strongly influences endothelial function. The distinction between endothelial activation and dysfunction is often times missing, and both concepts are frequently mixed. Since the endothelium is an “active” as opposed to “quiescent” tissue, constantly reacting to maintain vascular homeostasis, activation is not necessarily detrimental. However, endothelial dysfunction is always a pathological condition [39], that occurs when the normal functions of the endothelium shift towards reduced vasodilation, a pro-inflammatory state, and pro-thrombotic properties [23]. Cytokines like interleukins 1-β (IL-1β) and 6 (IL-6) or tumor necrosis factor alpha (TNF-α) activate the endothelium and lead to a decrease in NO synthesis and a VCAM-1 overexpression [40,41,42,43,44]. On the other hand, replacement of injured ECs may occur after the production and release of circulating endothelial progenitor cells from the bone marrow [42].

### 3.1. Nitric Oxide Production and Regulation

In normal conditions, the endothelium produces NO and prostacyclin, in response to physical stimuli, hormones, and platelet-derived substances. This results in vascular relaxation and platelet function inhibition [23]. NO is an important element in endothelial function, namely in vascular tone modulation, leukocyte adhesion regulation, vascular smooth muscle proliferation, and platelet aggregation [45]. Once produced it diffuses to vascular smooth muscle cells, where it activates guanylate cyclase, which, in turn, leads to increased production of cyclic guanosine monophosphate (cGMP), a reduction in intracellular calcium levels, and ultimately induces vasodilation [46,47]. NO also reacts with oxygenated Hb to produce metHb and nitrate (NO_3_^•−^), and with deoxygenated Hb to produce nitrosyl-Hb [48]. In conditions where high levels of cell-free Hb are present (e.g., hemolytic states, as in SCD) this leads to NO scavenging, and to decreased NO bioavailability and endothelial dysfunction.

NO is produced by nitric oxide synthases (NOS). NOS may occur as one of three isoforms: neuronal NOS (nNOS, or NOS1), inducible NOS (iNOS, or NOS2), and endothelial NOS (eNOS, or NOS3). Each has a specific tissue location and type of expression [49]. Endothelial NOS is constitutively expressed by ECs, hence it is fundamental for the regulation of endothelial NO bioavailability, but has also been detected in other cell types such as cardiac myocytes, platelets, neurons in the brain, syncytiotrophoblasts of human placenta, and LLC-PK_1_ kidney tubular epithelial cells [50]. It is upregulated by calmodulin, through Ca^2+^ mediation [51], heat-shock protein 90 (hsp90), estrogen, vascular endothelial growth factor (VEGF), bradykinin, and fluid shear stress [52,53,54].

On the other hand, eNOS may be downregulated by caveolin-1 (produced by caveolae of ECs) [55]. The physiological functions of NO, produced by eNOS, include vasodilation and inhibition of platelet aggregation and adhesion [56,57], inhibition of leukocyte adhesion and vascular inflammation [58,59], control of vascular smooth muscle proliferation, [60,61], stimulation of angiogenesis [59,62], and activation of endothelial progenitor cells. In enhanced oxidative stress states, like the ones present in cardiovascular diseases, increased NO degradation occurs due to its reaction with O_2_^•−^ [49]. Oxidative states also convert eNOS from an enzyme that synthesizes NO to an enzyme that produces O_2_^•−^—a condition called NOS uncoupling [63]. Other mechanisms that lead to eNOS uncoupling include oxidation of (6R-)5,6,7,8-tetrahydro-L-biopterin (BH4) (a critical eNOS co-factor) [64], L-arginine depletion [65], accumulation of endogenous methylarginines [66], and S-gluthationylation of eNOS [67,68].

### 3.2. Endothelial Dysfunction vs. Endothelial Activation

Endothelial dysfunction, particularly a reduced ability to synthesize NO and NO-mediated vasodilation, is common in patients with cardiovascular disease (e.g., coronary heart disease, atherosclerosis) and with risk factors for cardiovascular disease (e.g., hypertension, diabetes, obesity) [29,69,70].

Changes in the expression of surface adhesion molecules, leading to adhesion between SSRBCs and other blood cell types, promote multicellular aggregation, further contributing to vascular occlusion. Increased “adhesiveness” towards ECs is also apparent, especially in the microvasculature, causing an “arrest” of those aggregates inside the vessels, and contributing to proliferation of the endothelial layer and increase in-blood viscosity [71]. Furthermore, the overexpression of CAMs by the endothelium also results in shedding to the vascular lumen. High levels of soluble CAMs, especially VCAM-1, constitute strong biomarkers of endothelial activation and further contribute to progressive activation and proliferation. This ultimately results in vaso-occlusion and further intra- and extravascular hemolysis [48,71,72].

### 3.3. Chronic Inflammation and ROS Production

Several endothelial functions are susceptible to changes or impairment, which could lead to cell death and tissue injury. Inflammatory alterations are one cause of endothelial dysfunction already described in the pathogenesis of CVD [73,74].

Intracellular HbS polymerization increases inside RBCs under hypoxic (or acidic) conditions, and higher levels of polymerized HbS enhance RBC sickling. The cell membrane damage resulting therefrom causes an exposure of cell membrane elements, like phosphatidylserine (PS), and the production of ROS [75]. SSRBCs may cause endothelial injury, which, in addition to intravascular hemolysis, activates ECs, promoting the expression of pro-inflammatory signals, like endothelin-1 or the NF-κB pathway. Activation of NF-κB signaling triggers the upregulation of CAMs, such as E-selectin, VCAM-1, and ICAM-1 [48,76], which mediate leukocyte recruitment and adhesion [77]. Increased adhesive properties of SSRBCs, together with CAM overexpression, contribute not only to amplify adhesive interactions between SSRBCs and ECs but also with leukocytes and platelets. This stimulates a pan-cellular activation that culminates in an upregulation of cytokines, like TNF-α, granulocyte-macrophage colony-stimulating factor (GM-CSF), IL-1, IL-3, IL-6, IL-8, and macrophage colony-stimulating factor [41,44,78,79]. Additionally, high serum levels of the inflammatory modulators prostaglandins E1 and E2, as well as of the angiogenic growth factor, have been found in SCD patients.

The damaged membrane of SSRBCs makes them prone to premature cell death, which results in the release of Hb and its prosthetic moiety heme into the plasma. The cell-free Hb contributes to inflammation in SCD by scavenging NO, promoting oxidative stress, triggering apoptosis and endothelial barrier dysfunction [16,80,81]. Besides upregulating endothelial CAM expression, heme may also lead to the activation of circulating neutrophils and the formation of neutrophil extracellular traps in the vasculature, and subsequent organ injury [82]. Furthermore, studies in murine SCD models indicate that cell-free heme/hemin pro-inflammatory signaling is dependent on toll-like receptor-4 (TLR4) and NF-κB signaling [79,82,83]. Moreover, cell-free HbS, unlike HbA or heme, has been reported to elicit a significant enhancement in the expression of proinflammatory cytokines by human monocytes. This effect was found to be mediated by direct interaction with the TLR4/myeloid differentiation factor 2 (MD-2) complex, resulting in the activation of both the NF-κB and type I interferon pathways. Additionally, the authors found that in Townes SCD mice, injection of HbS, unlike HbA, was responsible for an increased production of proinflammatory cytokines, which was prevented by administration of TAK-242, a TLR4 inhibitor [84].

### 3.4. Intravascular RBC Hemolysis and Production of RNS

Together with inflammation, hemolytic anemia is also a chronic SCD complication which is more severe in SCA patients than any of the other SCD forms. Hb concentration varies, not only across SCD genotypes, but also among individuals with the same genotype. In SCA, the RBC survival range is two to twenty-one days. Clinical markers of hemolysis—total Hb concentration, reticulocyte count, bilirubin, and lactate dehydrogenase (LDH) levels—also mirror this variability [85,86,87,88,89].

Hemolysis results from SSRBCs’ fragility. It can occur inside or outside blood vessels (intra- or extravascular hemolysis, respectively). Chronic intravascular hemolysis results in the release of Hb, heme, and other RBC microparticles into the circulation. This strongly affects NO bioavailability and is a major underlying mechanism of several SCD complications. Cell-free Hb (oxyHb, Fe^2+^) binds to NO and rapidly converts it into bioactive NO_3_^•−^, shortening its half-life, and reducing its ability to diffuse across cell membranes. This process also produces metHb (containing Fe^3+^) [16,82]. Additionally, an arginase release depletes L-arginine, an amino acid critical for NO production. The subsequent decrease in NO bioavailability leads to vascular tone imbalance, platelet activation, and aggregation, as well as the transcriptional upregulation of VCAM-1, ICAM-1, P-selectin, and E-selectin [69]. Free Hb, heme, and heme iron catalyze the production of oxygen radicals. This further decreases NO endothelial availability and promotes endothelial dysfunction. It also disturbs the vascular tone and skews the balance towards vasoconstriction, endothelial activation, and proliferation [90].

### 3.5. Tissue Ischemia-Reperfusion Injury and ROS Production

Vaso-occlusion promoting events also contribute to the pathogenesis of tissue I/R injury, particularly microvascular dysfunction. I/R injury in SCA differentiates it from other chronic anemia states [91]. The resulting obstructed blood flow results in ischemic alterations in downstream tissues, exposing all ECs in the post-ischemic tissues to the same damaging effects. After the obstruction resolves, an inflammatory reaction causes local injury that may progress to systemic inflammation, damaging organs distal to the injury site, and potentially ending in life-threatening multi-organ injury or failure [92,93].

Studies in sickle murine models have demonstrated the importance of I/R injury in this disease [94,95]. Despite the common I/R insult, arterioles, capillaries, and venules react in a site-specific manner. Arterioles show a diminished acetylcholine endothelium-dependent vasodilation response [96,97]. In capillaries, the endothelial barrier function is impaired, resulting in interstitial edema, and leukocyte capillary plugging reduces the number of perfused capillaries, which enhances hypoxia [98]. Nevertheless, reperfused post-capillary venules are the vessels that bear the stronger impact of vascular response to I/R, especially through increased leukocyte–endothelial cell adhesion, platelet–leukocyte aggregation, excessive albumin extravasation, and higher oxidant production [99,100]. In SCD, vaso-occlusion precipitates the initial damage and causes local tissue hypoxia. Unable to undergo aerobic respiration, the oxygen-deprived cells become deficient in ATP, and, after mitochondrial dysfunction associated with intracellular hypercalcemia, they swell and undergo cell death [101].

Microvascular dysfunction due to I/R injury in SCD includes changes like microvascular permeability, pro-inflammatory and pro-coagulable EC activation, alterations in vasoactive mediator levels, and production of ROS [91]. Hypoxia causes necrosis, leading to increased levels of hypoxanthine, and to the conversion of xanthine dehydrogenase into xanthine oxidase (XO). Upon normalization of blood flow and oxygen delivery, those levels of hypoxanthine and XO become toxic [94,102,103]. NO deficiency also occurs as a result of inflammatory, hemolytic, and oxidant processes. Loss of NO signaling promotes activation of leukocytes, platelets, and NF-κB, while also contributing to the release of P-selectin and vWF from Weibel–Palade bodies [104].

Recurrent microvascular occlusions lead to persistent vascular damage and all organs are potentially affected by systemic I/R injury. However, animal studies indicate that different organs show different degrees of susceptibility to that damage, with the brain, heart, and kidney being more vulnerable to local ischemia and I/R injury [95].

## 4. Oxidative Pathways, Vasculopathy, and SCA

In SCA, oxidative stress results from the participation of several players in the altered redox biology of the disease, namely SSRBCs, leukocytes, monocytes, and the vascular endothelium. SSRBCs have high baseline concentrations of ROS, hydroxyl free radical (^•^OH), O_2_^•−^, and hydrogen peroxide (H_2_O_2_), when compared to normal RBCs, which suggests the presence of a pro-oxidant environment in SCA patients even before the onset of clinical manifestations [105]. Oxidant mediators may also take part in I/R injury and inflammation, which are frequent in SCA.

As stated above, one of the main mechanisms of oxidative stress in SCA is HbS autoxidation. Inside SSRBCs, HbS quickly oxidizes to form metHb, hemin, Fe^3+^, and O_2_^•−^. This affects several RBC activities, such as cytoskeletal oxidation, membrane lipid peroxidation, and PS exposure. Due to lack of mitochondria, RBCs use a glycolytic pathway (the Embder–Meyerhof pathway) to produce energy (ATP), which also produces reduced nicotinamide-adenine-dinucleotide (NADH). NADH is necessary for metHb reductase to reduce inactive metHb (containing ferric ions) to functionally active Hb (containing ferrous ions). In the case of SSRBCs, this mechanism allows metHbS reduction to its more innocuous ferrous state. However, SSRBCs also show a depletion in glutathione, an additional scavenger of ROS, thus enhancing oxidative stress in these cells [106]. In normal RBCs, the sidearm of the Embder–Meyerhof pathway (the Luebering–Rapoport shunt) generates 2,3-diphosphoglycerate (2,3-DPG), which is important in Hb-O_2_ affinity regulation [107,108]. In SSRBCs, 2,3-DPG levels (as well as sphingosine-1-phosphate, S1P) are increased, which reduces HbS-O_2_ affinity, and consequently leads to higher levels of deoxyHbS and further polymerization. Sickling and RBC oxidative damage lead to RBCs shedding microparticles. In turn, these have been shown to induce ROS production in cultured ECs, and cause vaso-occlusion in sickle mouse kidneys [109]. In SCD, PS exposure upon deoxygenation is linked to Ca^2+^ influx that causes Gardos channel activation and signals eryptosis (RBC cell death) [110,111]. Additionally, PS exposure increases adhesion (activating ECs and contributing to inflammation) and promotes coagulation (through direct platelet activation or by hemodynamic change at the vessel wall, indirectly leading to EC/platelet interaction) [112,113].

The constant supply of cell-free and heme/hemin provided by chronic hemolysis also promotes a pro-oxidant vascular environment. Besides the interactions between oxyHb with NO, it may also react with H_2_O_2_ through the Fenton reaction to form ^•^OH and metHb. The latter may further degrade into hemin, which is a major RBC damage-associated molecular pattern (DAMP) molecule. The hemolysis-related ROS contribute to the activation of inflammation and adhesion in endothelial cells, platelets, and neutrophils. This may ultimately result in vaso-occlusion, as demonstrated by a study by Ghosh et al. [114], where direct hemin infusion induced acute vaso-occlusive crises (VOC) in sickle mouse lungs, which was improved by inhibiting P-selectin pathways.

The oxidative damage effects are enhanced further in SCD due to the failure/downregulation of several antioxidant pathways or oxidant scavenger molecules. Hemolysis-related rapid clearing of haptoglobin and hemopexin, which scavenge cell-free Hb and hemin, respectively, ref. [115] is one example. Co-administration of haptoglobin and hemopexin was shown to restore microvascular blood flow in sickle mice that presented with acute vascular stasis due to infusion of exogenous Hb or hemin [79]. Furthermore, upregulated gene expression of hemopexin in hemin-infused Townes-SS mice (a SCA murine model) prevents microvascular occlusion, increases expression of the protective liver nuclear factor erythroid 2-related factor 2 (Nrf2) and heme-oxygenase-1 (HO-1) activity, as well as decreases pro-inflammatory NF-κB. On the other hand, venostasis is worsened in sickle cell hemopexin knock-out mice exposed to hemin [116]. Nrf2 activation has an oxidant protective effect in SCD, by removing hemolysis-derived free heme [117], and it has also been shown to induce fetal hemoglobin (HbF) by binding to γ-globin antioxidant response element [118].

HO-1 is the inducible isoform of the enzyme heme-oxygenase (HO), and its gene’s *HMOX1* expression is increased by the presence of heme, which the enzyme converts to biliverdin, carbon monoxide (^•^CO), and ferrous iron, along with several additional protective effects. Belcher and colleagues have shown that, in the Townes-SS murine model, a single co-infusion of hemopexin and haptoglobin upregulated HO-1 expression and lowered NF-κB activity in several tissues, offering protection from vaso-occlusion for up to 48 h [119]. The authors also suggested that the benefits of haptoglobin and hemopexin do not depend exclusively on rapid Hb and hemin clearance from circulation, since the effect of HO-1 on NF-κB activity and venostasis were independent of Hb and hemin plasma concentrations. In a subsequent study, using the same murine model, the administration of oral ^•^CO upregulated Nrf2 and HO-1, and downregulated NF-κB activity, soluble VCAM-1, and venostasis [120].

Other mechanisms that may further contribute to increased oxidative stress in SCD include high mobility group box 1 (HMGB1)-mediated TLR4 signaling, that activates the NF-κB pathway and may affect endothelial CAMs’ expression (e.g., P-selectin), and mitochondrial dysfunction in platelets, in addition to non-heme-dependent mechanisms [114,121,122]. The latter include NADPH oxidase, XO, and uncoupled eNOS, all of which generate oxygen free radicals, thus promoting endothelial dysfunction. Myeloperoxidase (MPO), generated by activated neutrophils, also produces oxidants that scavenge NO, further contributing to endothelial dysfunction [123,124]. Increased levels of the oxidative stress biomarkers MPO, HO^•^, lipid peroxidation, and total thiols were recently confirmed in SCD, while antioxidants superoxide dismutase (SOD), glutathione, and catalase levels were reduced [124,125].

## 5. Cerebrovascular Disease and Hypoxia in SCA

Since SCD is mainly a chronic vascular disease, the majority of its pathobiological mechanisms arise due to disturbance of the homeostasis inside the micro- and macro-vasculature. Although the core mechanism results from altered properties of SSRBCs and their interactions with other blood cells or with the vascular endothelial wall, several other mechanisms are also involved (Figure 3).

In SCD, vasculopathy arises as a combined multistep process that comprises, but it is not limited to, decreased NO bioavailability, oxidant I/R injury, elevated leukocyte count, platelet activation, and increased levels of multiple inflammatory mediators [126]. Therefore, vasculopathy is central to several of the clinical complications as it often takes place before end-organ dysfunction. SCD-related vasculopathies include, among others: moyamoya (that usually precedes cerebral infarcts/hemorrhage), proliferative retinopathy (prior to eyesight loss), pulmonary vasculopathy (associated with pulmonary hypertension), and renal vasculopathy (preceding chronic renal disease) [127].

CVD is a severe SCA complication, particularly in children, with stroke constituting a highly devastating manifestation [128]. Cerebrovascular accidents are among the more frequent clinical manifestations in children with SCA, together with acute pain crises (or vaso-occlusive crises, VOCs), splenic sequestration, recurrent infections due to functional asplenia, acute chest syndrome, cholelithiasis, nocturnal enuresis, hematuria, and reduced (and delayed) growth [129]. Pediatric neurological disease risk is high in SCD and may have significant consequences, especially in the case of overt stroke. Conversely, SCA is the most common cause of pediatric stroke, as children with SCA have a 300-fold increase in stroke risk. Moreover, stroke are is more frequent in SCA than in other SCD presentations [128]. High levels of HbF, as well as co-inheritance of α-thalassemia, are associated with milder SCD phenotypes, and are thus recognized as major disease modifiers [130]. However, this modulation only accounts for a small portion of the phenotypical heterogeneity observed.

Subclinical cerebral infarctions (or silent cerebral infarcts, SCI) are even more common but require magnetic resonance imaging (MRI) for diagnosis. These lesions correlate with marked neuropsychological deficits, and approximately half of the children with SCIs often require life-long support or custodial care [131]. Significant physical and cognitive deficits limiting quality of life are frequent after an overt stroke even if the child follows the recommended therapy [132]. As with other SCD complications, cerebrovascular manifestations are varied—ranging from extensive, large vessel distributed infarcts, to more subtle lacunar infarcts—and differ in terms of epidemiology, clinical features, and pathology [133]. The risk of cerebrovascular complications is highly increased in SCA, predisposing patients to ischemic or primary hemorrhagic stroke [128,134,135]. Pediatric stroke and SCIs occur at a high cumulative rate in SCA [128,136]. A 20-year-old SCA patient has an 11% probability of having already experienced at least one stroke event, and this risk increases to 24% by age 45 [128]. Whereas ischemic stroke is the more common form in young children, intracranial hemorrhage tends to occur later in life [128]. SCIs are even more frequent, with about 37% of children suffering at least one event before reaching 14 years of age. Furthermore, children that have had previous SCIs are at increased risk of developing stroke.

Brain imaging is fundamental for confirming overt strokes (clinically apparent and with an abrupt onset of neurological manifestations) and diagnosing SCIs (present on brain MRI, but without clinical symptoms, or external signs that correlate to the neuroimaging findings) [136]. Cerebral vasculopathy is a major underlying mechanism of ischemic stroke, according to data obtained by cerebral angiography or magnetic resonance angiography (MRA) [137,138,139]. Concomitantly, the most common histopathological finding in SCD-related cerebral vasculopathy is endothelial damage in the mid- to large-sized brain arteries [140,141]. That damage occurs particularly in the branch points, and infarcts in SCA patients are more frequent where stenosis and occlusion occur, particularly in the area of the bifurcation between the internal carotid artery and the middle cerebral artery—the Circle of Willis. The infarct distribution in histopathological studies is consistent with the hypothesis that overt strokes in SCD are a consequence of mid- to large-vessel disease, affecting mainly the distal internal carotid, proximal middle cerebral, and anterior cerebral arteries [142,143].

The lower oxygen affinity of HbS and its polymerization under hypoxic conditions trigger a cascade of events from chronic hemolysis, to microvessel occlusion, endothelial activation and dysfunction, inflammation, and I/R injury. Tissue ischemia in the brain results in ischemic stroke, which in turn tends to occur in the border zones, or watershed regions, even in the absence of large-vessel vasculopathy [144,145,146].

In non-SCD ischemic stroke patients, arteriolar dilation occurs to maintain cerebral blood flow (CBF), and increases in oxygen extraction fraction (OEF, the fraction of oxygen extracted from blood by the brain tissue) contribute to keep the cerebral metabolic rate of O_2_ utilization (CMRO_2_) under conditions of decreasing perfusion pressure [147,148]. When these compensatory mechanisms are insufficient to meet the tissue’s metabolic demands, lowering the CMRO_2_, the result is a cerebral infarct [149]. In those adult patients who have carotid occlusive disease, elevated hemispheric OEF may be a robust indicator of stroke risk [150].

CBF is elevated in children with SCD [146,151], a compensatory mechanism for chronically low arterial oxygen content (oxygen-carrying capacity) due to anemia. Another study proposed that elevated OEF in the deep white matter reflects a metabolic stressed cerebral tissue, and is associated with increased stroke risk in those children [146]. Cerebral regional changes in CBF, OEF, and CMRO_2_ were observed in the deep white matter of pediatric SCD patients, with OEF peaking when CBF and CMRO_2_ are at their minimum. This was consistent with a failure to meet metabolic demand in regions of lower CBF and CMRO_2_, despite high OEF, as these were areas with higher infarct density. The overlap between areas of high infarct density and high OEF, but without low CBF, indicated that high OEF is a stronger indicator of stroke risk than low CBF [146]. Furthermore, a subsequent study showed that children with SCD cerebral metabolic stress may have their symptoms ameliorated with hydroxyurea (HU) therapy (see below). That strategy could be an adjuvant to the most used imaging tool for stroke risk prediction—the transcranial Doppler ultrasonography for measuring the time-averaged mean of maximum velocity (TAMMV) in the middle cerebral artery. This quantitative assessment of stroke risk relies on the Bernouilli’s principle of fluid dynamics. Briefly, this principle states that the speed of a fluid increases when there is a decrease in pressure. In the case of cerebral vasculopathy, that decrease takes place distally to the region where a partial occlusion (or narrowing) of the blood vessels occurs. Hence, if one measures the blood flow velocity in the large arteries mentioned earlier, this could be a strong indicator of the occurrence of a vaso-occlusive or stenotic event.

Children with SCA have a high prevalence of SCIs and these are the most frequent form of SCA-related neurological injury. They may appear very early in life and remain undetected, unless the child undergoes a brain-imaging exam, or until an overt stroke occurs. As mentioned previously, SCI risk is cumulative; up to 37% of children with SCA have suffered an SCI by age 14 years, and this increases to up to 53% in adults by the age of 32 years [128,136,152]. In addition to brain injury, SCI events are associated with increased overt stroke risk [138], failure to meet academic milestones [131], and increased cognitive impairment [132,153,154].

Contrary to strokes, SCIs do not seem to result from macrovasculopathy. Initially, studies suggested that they were spatially circumscribed to the white matter of the frontal and parietal lobes [138]. Subsequent studies demonstrated the occurrence of white matter loss [155] and disrupted matter integrity [156], as well as border zone [155,157] and cortical wedge-like infarcts [158]. The occurrence of border zone infarcts suggests that ischemic mechanisms may result from a global reduction in arterial oxygen content, cerebral hemodynamic factors, or both, while wedge-like infarcts indicate a predominance of thromboembolic factors [158]. With no apparent external manifestations, SCI diagnosis relies heavily on brain imaging technology, like MRI. A large prospective study with a pediatric SCA cohort showed that CBF decreased as infarct density increased, thus confirming that SCIs predominantly occur in the border zone vascular distribution within the white matter of the frontal and parietal lobes [158]. Additionally, the results demonstrated that 90% of children had SCIs within a relatively small border zone region, measuring 5.6% of total brain volume, and they occurred in the region of low blood flow.

## 6. Antioxidant Therapeutic Approaches in SCD Vasculopathy

In general, current SCA treatments target symptom relief and, where possible, primary prevention. Even though an established prevention therapy is in place for pediatric stroke prevention in high-income settings, the challenges for CVD treatment are similar to those for other SCA manifestations. Nevertheless, achieving a one-size-fits-all therapeutic solution is highly unlikely, due to the complex nature of SCA. While upstream-targeted strategies focus on the β-globin gene cluster expression, namely the Hb switching mechanism, downstream-targeted approaches address the multitude of pathophysiological mechanisms of SCD. Upstream approaches are recent and rely on circumventing the genetic defect, whether directly (by correcting the mutation) or indirectly (by reversing the physiological switch in Hb production).

Current approaches to genetic-based therapies involve collecting a patient’s hematopoietic stem cells (HSC) via bone marrow harvesting or apheresis. This is followed by genetical modification of the cells ex vivo by inserting a new transgene, or editing the gene directly. Finally, the genetically modified progenitor cells are reinfused after high-dose chemotherapy conditioning. Despite the advantages of not requiring donors, thus reducing the risk of graft-vs-host disease [159], these strategies are highly dependent on several key issues. These include: quality of viral delivery systems; quality and quantity of HSC harvested; optimization of the gene modification system in HSC; choice of recombination pathway (homologous vs. non-homologous); identification of the best gene targets; cell manufacturing; preparation regimens to allow the bone marrow to receive genetically modified cells with minimized toxicity; issues related to off-target effects; optimization of preclinical models for testing of developing gene therapy strategies; and parameters that should be used to define a cure [160].

Downstream strategies are especially challenging due to the high complexity of SCD where much is still unknown. In terms of pharmacological disease-modifying treatments, and despite numerous recent clinical trials, the European Medicines Agency (EMA) and the Food and Drug Administration (FDA, USA) have approved only four substances—HU, L-glutamine, voxelotor, and crizanlizumab.

Hydroxyurea was the first drug approved for treatment and acts on a variety of mechanisms involved in SCD. Those mechanisms include erythroid regeneration, NO-related increase in the activity of soluble guanylate cyclase and cyclic guanidine monophosphate (cGMP), which stimulates HbF-related genes expression, or NO increase through long-term post-transcriptional rise in eNOS levels [161,162]. The increase in HbF production counteracts the destructive repercussions of HbS presence, thus acting as a disease modifier. This translates into several beneficial effects in SCD, such as lower incidence of pain episodes and acute chest syndrome, as well as a reduction in the number of hospitalizations [163,164]. Furthermore, HU decreases mortality and morbidity associated with VOC events [165], cerebrovascular accidents [152,166], and proteinuria [167], thus increasing overall survival in patients with SCD [168]. This is especially important in low-resource settings where blood transfusions are not easily available, and/or are less equipped to manage the downsides of long-term transfusion therapy (e.g., risk of alloimmunization or iron overload, the need for a robust antigen matched blood supply). Therefore, HU has a similar effect on TCD velocities as blood transfusion therapy in children with SCD at high risk of stroke [164,166]. A recent study demonstrated that HU scavenges free radicals and induces the expression of antioxidant genes [169].

Of the remaining approved drugs for SCD treatment, only L-glutamine was shown to have an effect on ox–redox vascular balance. It is a NAD precursor, which provides a supplementation that will counteract NAD depletion on SSRBCs [170]. The drug has been described to reduce acute complications of SCD, both in pediatric (<5 years of age) and adult patients, namely VOC painful crises and hospitalizations [170]. Despite rapid approval, much is still unknown regarding the L-glutamine mode of action.

Voxelotor’s mode of action has been assessed in studies like the Hemoglobin Oxygen Affinity Modulation to Inhibit HbS Polymerization (HOPE) clinical trial [171]. It acts as an inhibitor of HbS polymerization, thus stabilizing HbS in the oxygenated state and inhibiting RBC sickling. Voxelotor was shown to increase Hb levels, thus reducing the number of acute anemia episodes. Levels of hemolysis markers (namely bilirubin, but also reticulocyte and LDH) were also decreased. The HOPE study did not measure stroke incidence, despite initial concerns that increasing oxygen affinity would reduce oxygen extraction in sensitive tissues like the brain [159,172].

Crizanlizumab (SelG1) is a humanized anti-P-selectin antibody that, in a study to assess safety and impact of SelG1 with or without HU therapy in SCD patients with pain crises (SUSTAIN), was shown to be effective in reducing VOC frequency and acute chest syndrome [173]. The rationale for its application was to decrease both RBC and leukocyte adhesion, which occurs by downregulating P-selectin expression and consequently inflammation-mediated cell adhesion. However, CVD was not a primary or secondary endpoint in the SUSTAIN study.

Research on further pharmacological targets is ongoing. It relies heavily on further knowledge of SCD pathobiological mechanisms. The most promising target mechanisms for pharmacotherapy identified so far, besides HbF-stimulating agents, include adhesion, I/R injury, coagulation, and hemolysis [173,174,175,176,177,178,179]. To date, and besides HU, statins like simvastatin are the ones that, besides an anti-inflammatory mode of action, also seem to act on eNOS to restore nitric oxide production [180].

Other antioxidant agents are potential adjuvants for SCD therapy though none of them have yet been approved by the FDA or the EMA. These include N-acetylcysteine, zinc supplementation, nitric oxide, L-arginine, α-lipoic acid, and acetyl-L-carnitine [13,181].

Given the multitude of effects caused by SSRBCs, the more adequate therapeutic strategy will probably be a multitargeted approach [182]. Unfortunately, the limited number of SCD patients in high-income countries and scarce resources in low-income countries make it difficult to accomplish definitive studies of many pharmacological agents [183].

## 7. Genetic Modulation of Cerebrovascular Disease in SCA

The standard of care in SCD pediatric stroke prevention is TCD screening, followed by regular blood transfusion therapy for children identified as being at high risk. The definition of risk depends on the TAMMV values, as follows: high risk TAMMV > 200 cm/s; moderate risk 70 cm/s < TAMMV < 200 cm/s [134]. Unfortunately, the relationship between TCD velocities and stroke incidence is not precise, nor age-independent. Approximately seven children with elevated TCD value have to be treated with regular blood transfusion to prevent one child from having a stroke [127]. Stroke risk also seems to be age-dependent, as individuals with ages above 16 years and elevated TAMMV do not appear to have a significant increase in stroke risk [184]. Furthermore, the management of hematological disease alone does not seem to prevent vasculopathy progression, which indicates that additional genetic risk factors have a role as risk modulators. This is especially important since heterogeneity in disease presentation and severity complicates prognostication, management, and clinical trials [185].

Several biomarkers of cerebral vasculopathy were already identified, including low HbF level [186], low baseline Hb, high leukocyte count, male sex, and relative high systolic pressure [128,187]. Moreover, high levels of HbF and co-inheritance of α-thalassemia are recognized modifiers of global disease severity [2,188]. Several studies indicated that gene variants co-inherited with the β^S^ mutation are potential modifiers of stroke/stroke risk [189,190,191,192,193,194,195,196].

Research approaches that ranged from candidate genes to genome-wide association studies (GWAS) focused on identifying potential stroke/stroke risk modifying loci [193,194,197,198,199]. Those genetic modifiers, or modulators, could be potential targets for increasingly accurate (and possibly personalized) prognostic tests and therapeutic strategies. The results indicated that, besides the previously demonstrated protective role of the co-inheritance, the most powerful genetic modifiers of disease severity lie within the β-globin cluster [200]. Identification, confirmation, and functional assessment of other genes requires knowledge of their involvement in stroke pathophysiology. Several putative stroke-associated variants were identified in genes such as *VCAM1* [191,193,196], *IL4R* [193], *LDLR* [193], *ADRB2* [193], *AGT* [190], *HLA* [189], and *TNF*-α [193,195], as well as the -α^3.7kb^ deletion that causes α-thalassemia [195] (Table 1).

Additionally, three SNPs identified by GWAS showed special significance: rs662 (Q192R) in the paraoxonase 1 gene (*PON1*), rs1044498 (K173Q) in the ectonucleotide pyrophosphatase/phosphodiesterase 1 (*ENPP1*) gene, and rs3732410 (Y1212C) in the Golgin subfamily B member 1 (*GOLGB1*) gene [198]. Of these, only the SNP on *PON1* was previously associated with increased stroke risk in adults [201,202]. The SNP in *ENPP1* was validated in another study [194]. On the other hand, our group identified a positive association between the presence of rs1044498_A allele and increased stroke risk [196]. We have also identified haplotypes of *VCAM1* promoter variants as well as a specific variant (rs1409419_T) with a strong association, not only with stroke [196], but also with hemolysis [203].

In terms of NO metabolism, we found a positive association with a *NOS3* gene variant with red cell distribution width (RDW) levels. Specifically, CC and TC genotypes of the rs2070744 showed a positive association with lower RDW (reduced anisocytosis) [196], a potential biomarker of lower cerebrovascular disease risk in non-SCD patients [204]. Another *NOS3* variant, a variable number of tandem repeats (VNTR) on the intron 4 specific allele with five repeats of 27 bp, also showed a potential protective effect against SCIs in the pediatric group of SCA patients that we studied [196].

**Table 1:** Candidate genes and respective variants previously identified as putative modifiers of stroke in sickle cell anemia.

Gene	Variant	Predicted Modulation	Reference
*VCAM1*	G1238C	Stroke protection	[191]
*VCAM1*	T1594C	Increased small-vessel stroke risk	[193]
*VCAM1*	rs1409419_T Haplotype 7	Increased stroke risk Increased stroke risk	[196]
*NOS3*	intron 4_27 bp VNTR_4b Haplotype V Haplotype VII	Decreased SCI risk Decreased SCI risk Decreased CV risk	[196]
*ITGA4*	rs113276800_CA rs3770138_T	Increased stroke risk Increased stroke risk Increased CV risk	[196]
*IL4R*	S503P	Increased large-vessel stroke risk	[193]
*LDLR*	Ncol +/−	Increased small-vessel stroke risk	[193]
*ADRB2*	Q27E	Increased large-vessel stroke risk	[193]
*AGT*	GT repeats	Increased stroke risk	[190]
*HLA*	DRB1*0301 DRB1*0302 DQB1*0201 DRB1*1501 DQB1*0602 DPB1*0401 DPB1*1701 -A*0102 -A*2612 -A*3301	Increased stroke risk Increased stroke risk Increased stroke risk Decreased stroke risk Decreased stroke risk Increased small-vessel stroke risk Increased small-vessel stroke risk Increased large-vessel stroke risk Increased large-vessel stroke risk Decreased large-vessel stroke k	[189,205]
*TNF-α*	-308G>A	Increased stroke risk	[193,195,206]
*GOLGB1*	Y1212C	Decreased stroke risk	[198]
*ENPP1*	K173Q	Decreased stroke risk Increased stroke risk Increased stroke risk	[198] [194] [199]
*PON1*	Q192R	Increased stroke risk	[198]
*HBA*	-α^3.7kb^ del	Decreased stroke risk	[195]

SCI—silent cerebral infarction; CV—cerebral vasculopathy; *VCAM1* haplotype 7–rs1409419_T/rs3917024_C/rs3917025_CT/rs3783598_T/rs1041163_T/rs3783599_C; *NOS3* intron 4_27 bp VNTR_4b–5 repeats × 27 bp; *NOS3* haplotype V–rs2070744_T/VNTR_4b/rs1799983_G; *NOS3* haplotype VII–rs rs2070744_T/VNTR_4b/rs1799983_T.

Assessing the biological role of the genetic variants potentially associated with SCD modulation can only be performed through functional studies. In SCD, these type of studies often rely on murine or cell models. Both types have provided important information regarding oxidative stress pathways, for example [169,196,207,208]. Studies on sickle mice used hemin (ferric heme) to simulate a sudden increase in intravascular hemolysis and blood flow stasis in post-capillary veins, due to its pro-oxidant and pro-inflammatory effects [79,209]. Heme-associated effects included the activation of the TLR-4 and NF-κB signaling pathways, that induced inflammation and vaso-occlusion, and the extent of subsequent damage was strongly associated with the presence of HbS [79]. Hemin injections also upregulated HO-1 expression. This, in turn, inhibited inflammation and adhesion.

Our studies showed that, in hemin-treated macrovascular and brain microvascular EC models, TNF-α-induced *VCAM1* expression was downregulated, and *HMOX1* was significantly upregulated [210]. Other authors reported that HO-1 production is also upregulated by hemin in monocytes, where it has an antioxidant effect by inhibiting apoptosis [207,208]. Furthermore, the results we obtained showed that both microvascular and macrovascular ECs present a similar *HMOX1* upregulation, thus suggesting little difference in the levels by which this antioxidant system operates in the macrovascular and brain microvascular environments. *HMOX1* expression also seems to be unaffected by HU not only in micro and macrovascular ECs [210] but also in peripheral blood monocytes (PBMCs) and umbilical cord vein ECs (HUVECs) [169]. This response is the opposite of what occurs with other antioxidant pathway genes’ expression, like superoxide dismutase (SOD), glutathione peroxidase (GPX), and glutathione-disulfide reductase (GSR), which were shown to be upregulated after HU treatment [169]. These observations indicate that *HMOX1* activation follows an alternative mechanism of antioxidant response not susceptible to HU action.

In addition, in the endothelial setting, the cytokine TNF-α leads to reduced NO bioavailability. This results from activation of endothelial arginase (which depletes eNOS from its substrate arginine) [211] and endothelial NADPH oxidase (lowering BH4 and leading eNOS to generate superoxide) [212]. In our research, we found that *NOS3* expression was downregulated in TNF-α-stimulated macrovascular ECs, and this effect was stronger in brain microvascular ECs. HU modulation of *NOS3* expression seems to be differential and dose-dependent [210,213], which indicates that HU effect in NO bioavailability does not result from direct *NOS3* upregulation. As proposed by several authors, the most likely mechanism by which HU increases eNOS levels is a post-translational proteasomal protection from degradation [162,213].

## 8. Conclusions

A clear understanding of the specific mechanisms and genetic variants modulating SCA manifestations is invaluable. This is particularly important for CVD, namely in children, due to its impact on patients’ lives. The possibility of defining a genetic marker panel associated with disease severity would allow for risk stratification of patients—together with the ability to provide timely care to high-risk patients and the identification of therapeutic targets, thus facilitating the design of new pharmacological agents, and the possibility of customizing therapeutic strategies to each patient in a precision medicine approach.

## Data Availability

Not applicable.
